# Association between hospital mortality and inspiratory airway pressures in mechanically ventilated patients without acute respiratory distress syndrome: a prospective cohort study

**DOI:** 10.1186/s13054-019-2635-y

**Published:** 2019-11-21

**Authors:** Sarina K. Sahetya, Christopher Mallow, Jonathan E. Sevransky, Greg S. Martin, Timothy D. Girard, Roy G. Brower, William Checkley, Society of Critical Care Medicine Discovery Network Critical Illness Outcomes Study Investigators

**Affiliations:** 1 Division of Pulmonary and Critical Care, Johns Hopkins University, 1830 E Monument St Room 555, Baltimore, MD 21287 USA; 2 Division of Pulmonary, Allergy, Critical Care, and Sleep Medicine, Emory University, Atlanta, USA; 3 Grady Health System, Atlanta, GA USA; 4 Clinical Research, Investigation, and Systems Modeling of Acute Illness (CRISMA) Center, Department of Critical Care Medicine, University of Pittsburgh School of Medicine, Pittsburgh, USA

**Keywords:** Driving pressure, Mechanical ventilation, Acute respiratory failure, ARDS

## Abstract

**Background:**

Higher inspiratory airway pressures are associated with worse outcomes in mechanically ventilated patients with the acute respiratory distress syndrome (ARDS). This relationship, however, has not been well investigated in patients without ARDS. We hypothesized that higher driving pressures (ΔP) and plateau pressures (Pplat) are associated with worse patient-centered outcomes in mechanically ventilated patients without ARDS as well as those with ARDS.

**Methods:**

Using data collected during a prospective, observational cohort study of 6179 critically ill participants enrolled in 59 ICUs across the USA, we used multivariable logistic regression to determine whether ΔP and Pplat at enrollment were associated with hospital mortality among 1132 mechanically ventilated participants. We stratified analyses by ARDS status.

**Results:**

Participants without ARDS (*n* = 822) had lower average severity of illness scores and lower hospital mortality (27.3% vs. 38.7%; *p* <  0.001) than those with ARDS (*n* = 310). Average Pplat (20.6 vs. 23.9 cm H_2_O; *p* <  0.001), ΔP (14.3 vs. 16.0 cm H_2_O; *p* <  0.001), and positive end-expiratory pressure (6.3 vs. 7.9 cm H_2_O; *p* <  0.001) were lower in participants without ARDS, whereas average tidal volumes (7.2 vs. 6.8 mL/kg PBW; *p* <  0.001) were higher. Among those without ARDS, higher ΔP (adjusted OR = 1.36 per 7 cm H_2_O, 95% CI 1.14–1.62) and Pplat (adjusted OR = 1.42 per 8 cm H_2_O, 95% CI 1.17–1.73) were associated with higher mortality. We found similar relationships with mortality among those participants with ARDS.

**Conclusions:**

Higher ΔP and Pplat are associated with increased mortality for participants without ARDS. ΔP may be a viable target for lung-protective ventilation in all mechanically ventilated patients.

## Background

Mechanical ventilation can increase survival rates of patients with acute respiratory failure. This life-saving technology, however, may also contribute to or worsen underlying lung injury through alveolar overdistention or repetitive opening and closing of small bronchioles and alveoli [1, 2]. In patients with acute respiratory distress syndrome (ARDS), a lung-protective ventilation strategy aimed at preventing overdistention injury by limiting tidal volumes and plateau pressures (Pplat) improves survival and is therefore recommended in clinical practice guidelines [3, 4]. Although these guidelines are frequently extrapolated to mechanically ventilated patients without ARDS, little evidence exists regarding the best ventilatory strategies for patients without ARDS.

Recently, driving pressure (ΔP) emerged as a potential target for optimizing mechanical ventilation to improve outcomes for ARDS patients. ΔP is calculated as the difference between Pplat and positive end-expiratory pressure (PEEP) and is determined by the ratio of the tidal volume to the compliance of the respiratory system (ΔP = Pplat − PEEP = V_T_/C_RS_). In patients with ARDS, lower ΔP has been associated with lower mortality in multiple studies and may be a critical mediator for the benefits of lung-protective ventilation strategies [5–8]. In patients without ARDS, lower ΔP was associated in one study with a decreased risk of postoperative pulmonary complications for patients with normal lungs [9]. However, studies evaluating the relationship between airway pressures and mortality in patients without ARDS are few and demonstrate inconsistent results [9–11].

Inspiratory airway pressures (Pplat and ΔP) are utilized as surrogates for lung stress during mechanical ventilation, and high levels of stress applied to the lung may be injurious even in patients who do not have ARDS. We hypothesized that higher ΔP and Pplat are associated with higher hospital mortality in non-ARDS patients as well as ARDS patients. To evaluate these relationships, we performed a secondary analysis of a large multicenter, prospective observational cohort of critically ill patients in the USA.

## Methods

### Study design

The Society of Critical Care Medicine (SCCM) Discovery Network Critical Illness Outcomes Study (CIOS) was a multicenter, prospective, observational cohort study of patients admitted to intensive care units (ICUs) in the USA. The content and protocol of this study have been previously described [12]. Eligibility criteria included adults ≥ 18 years of age physically occupying a bed at 8 a.m. in a participating ICU. Participants were enrolled in the cohort between November 2008 and January 2012 across 59 ICUs in the USA. Participating sites enrolled all patients in the ICU during one assigned day per week. Enrollment days were chosen randomly, with 5–10 days between enrollments to allow for patient turnover. Patients in the ICU who were present during prior enrollment days were not enrolled a second time. Due to this recruitment strategy, baseline participant data were recorded from the day of enrollment into this study rather than the first day of mechanical ventilation. Participants were enrolled from three different types of ICU: medical, surgical, and mixed medical-surgical. Data were prospectively recorded in detail including ICU structure and process variables, patient demographics, ventilator settings, and severity scores including APACHE II scores. Participants were followed until hospital discharge. The primary outcome measure for the CIOS study was hospital mortality. Secondary outcomes included duration of mechanical ventilation and duration of intensive care unit and hospital stay. The CIOS study was approved by the Institutional Review Boards at all participating hospitals (NA_00026710).

We analyzed all participants receiving mechanical ventilation on the day of enrollment into the cohort except for those who were missing measurements of Pplat, PEEP, or ARDS status. ARDS status on day of enrollment was based on the American European Consensus Conference (AECC) definition [13]. The primary exposure variable was ΔP on the day of enrollment into the study [5]. Daily ventilator settings were recorded at 8 a.m. on the day of enrollment. Ventilator pressures were collected on participants at the time of enrollment. ΔP was calculated as Pplat minus PEEP. Set PEEP was directly recorded from the ventilator. Pplat was measured by a minimum 0.5 s inspiratory hold maneuver at zero flow in participants on volume-controlled ventilation or as an estimation from the observed peak inspiratory pressure (PIP) in participants on pressure-controlled modes. We included participants on spontaneous breathing modes of ventilation such as pressure support given recent literature suggesting that Pplat and ΔP may be reliably estimated in the presence of spontaneous breathing [14, 15]. A sensitivity analysis evaluating the effect of including participants on spontaneous mode of ventilation was performed as described below. We excluded participants with biologically implausible values for Pplat, such as those with Pplat less than PEEP or with Pplat ≤ 5 cm H_2_O. The primary outcome was mortality at hospital discharge.

### Biostatistical methods

The primary objective was to evaluate the relationship between inspiratory airway pressures (Pplat and ΔP) and hospital mortality in participants without ARDS and to compare the relationship in participants with ARDS. We estimated the association between hospital mortality and inspiratory airway pressures (Pplat and ΔP) with multivariable logistic regression and used general estimating equations with a compound symmetry matrix and a robust variance to account for ICU-level clustering. For the multivariable analysis, we identified covariates that may be associated with mortality, could influence ventilator management, and were not collinear with ΔP. We did not include tidal volume or respiratory system compliance in regression models containing ΔP given concerns for collinearity as ΔP is determined by the ratio of tidal volume to compliance. Individual covariates included sex, age, Acute Physiology and Chronic Health Evaluation II (APACHE II) score, need for vasopressors, presence of sepsis, and PEEP at time of enrollment. ICU level covariates included type of ICU (medical, surgical, or mixed) and hospital volume (categorized as < 25,000, 25,000–39,999, or > 40,000 admissions per year). Odds ratios (OR) were scaled to the interquartile range (IQR) of the analyzed airway pressure.

We performed a secondary analysis evaluating Pplat as the primary exposure variable rather than ΔP. We evaluated this model to determine if both inspiratory airway pressures were associated with mortality for participants with acute respiratory failure regardless of ARDS status. We tested interactions between inspiratory airway pressures and ARDS status as well as inspiratory airway pressures and PaO2/FiO2 ratio using cross-product terms (e.g., ΔP×ARDS status), and used the likelihood ratio test to determine if including the cross-product term improved the model fit.

Multiple sensitivity analyses were conducted to evaluate the robustness of our results. First, we restricted the analysis to participants with Pplat less than 35 cm H_2_O to determine if our results were robust to participants without outlying Pplat and ΔP measurements. Second, we conducted a sensitivity analysis where we restricted our data to participants with PEEP ≥ 5 cm H_2_O. Third, we evaluated if the association between ΔP and hospital mortality was primarily related to the presence of hypoxemic respiratory failure (PaO_2_/FiO_2_ < 300 mmHg) in non-ARDS participants. Finally, we limited the analysis to participants on controlled mechanical ventilation modes (i.e., volume control, pressure control, synchronized intermittent mandatory ventilation, pressure-regulated volume control) to determine if including participants on spontaneous modes affected the results. Participants with missing data in either the primary outcome or the primary exposures were excluded from multivariable analysis. Missing data for included variables in our sample population of mechanically ventilated participants were less than 3% and were assumed to be missing at random.

For other analyses, continuous variables are presented as means (standard deviation) if normally distributed and medians (interquartile range) if non-normally distributed. Categorical variables are presented as counts (*n*) and percentages. Comparisons between variables were conducted using the Student *t* test for continuous variables, and the Pearson chi squared or Fisher’s exact test for categorical variables. We conducted statistical analyses in R (www.r-project.org) and STATA version 14.0 (College Station, TX). We analyzed and reported this study according to the Strengthening the Reporting of Observational Studies in Epidemiology (STROBE) guidelines.

## Results

### Participant characteristics

**Fig. 1:**
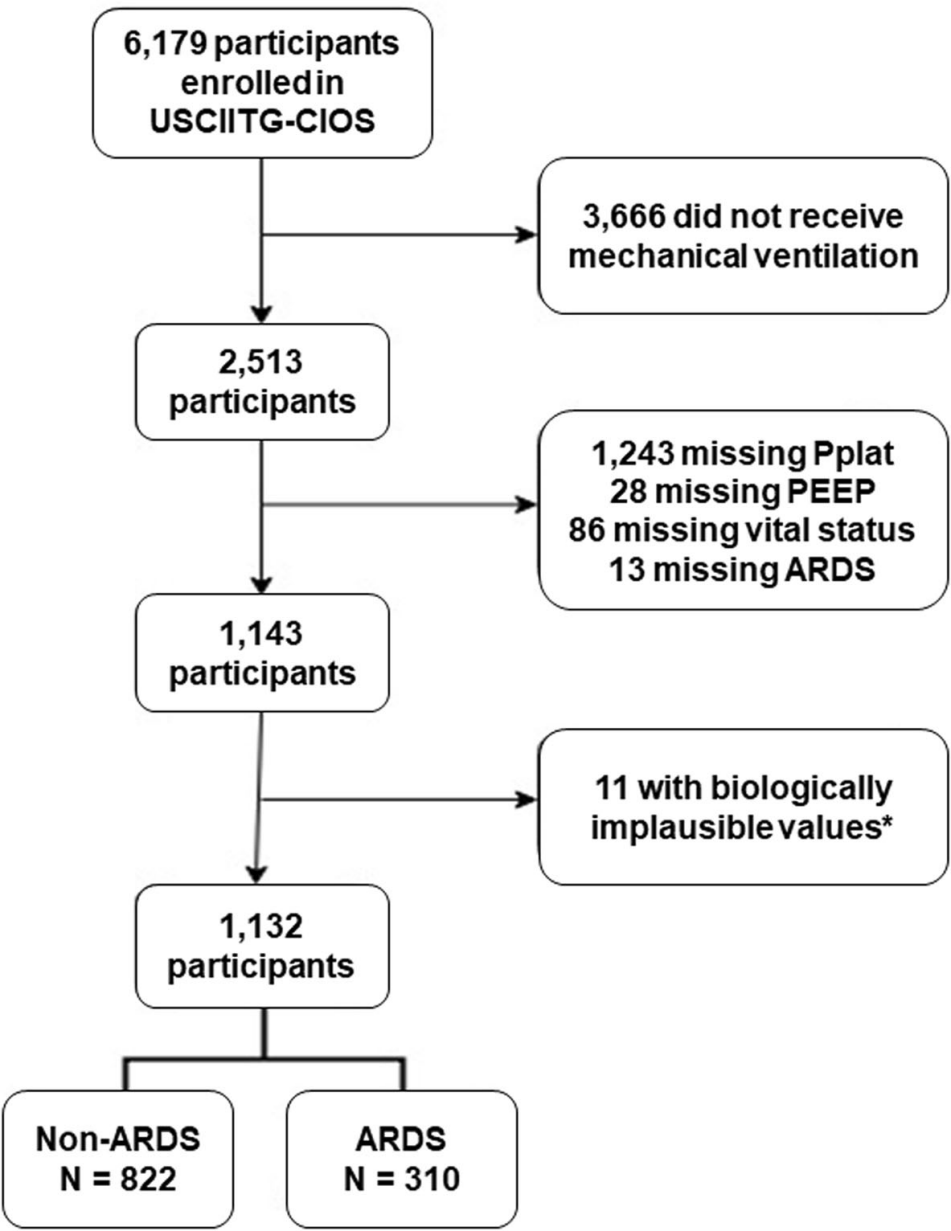
Flowchart of participants included in analysis. *Biologically implausible values defined as plateau pressure less than PEEP (*n* = 5) or plateau pressure less than 5 cm H_2_O (*n* = 6)

We enrolled 2513 mechanically ventilated participants from November 2008 until January 2012 in 59 ICUs. Of these participants, 1132 were eligible for analysis in our study (Fig. 1). Of these, 822 (72.6%) did not have ARDS and 310 (27.4%) had ARDS. Baseline characteristics of participants with and without ARDS were mostly similar (Table 1). However, participants without ARDS had lower severity of illness based on APACHE II (20.2 vs. 22.1) and SOFA scores (6.6 vs. 8.3 points). The primary reason for ICU admission was for a respiratory indication (non-ARDS 50% vs. ARDS 74%) with a high proportion of pneumonia in both groups (non-ARDS 30.4% vs. ARDS 47.4%). Mortality was lower in the non-ARDS group (27.3%) versus the ARDS group (38.7%).

**Table 1:** Baseline characteristics of non-ARDS and ARDS participants

	Non-ARDS (*n* = 822)	ARDS (*n* = 310)	*p* value
Age (years)	60.3 (16.6)	59.1 (15.9)	0.23
Male	440 (53.5)	170 (54.8)	0.69
African-American	209 (25.4)	61 (19.7)	0.04
Medical ICU	375 (45.6)	164 (52.9)	< 0.001
Initial admitting diagnosis^a^			
Respiratory	416 (50.6)	230 (74.2)	< 0.001
Neurological	251 (30.5)	48 (15.5)	< 0.001
Cardiovascular	226 (27.5)	84 (27.1)	0.89
Infectious	221 (26.9)	138 (44.5)	< 0.001
Gastrointestinal	122 (14.8)	44 (14.2)	0.78
Trauma	61 (7.4)	9 (2.9)	0.005
Endocrine	38 (4.6)	12 (3.9)	0.58
Other	134 (16.3)	37 (11.9)	0.067
Sepsis	261 (31.8)	190 (61.7)	< 0.001
Pneumonia	250 (30.4)	148 (47.7)	< 0.001
APACHE II	20.2 (7.4)	22.1 (7.7)	< 0.001
SOFA	6 (4–9)	8 (5–11)	< 0.001
PaO_2_/FiO_2_	255.6 (150.7)	174.8 (102.3)	< 0.001
Compliance respiratory system	39.6 (28.2)	35.1 (35.6)	0.04
Plateau pressure	20.6 (6.5)	23.9 (7.1)	< 0.001
Driving pressure	14.3 (6.0)	16.0 (6.4)	< 0.001
PEEP	5 (5–8)	7 (5–10)	< 0.001
Tidal volume (mL/kg PBW)	7.2 (1.21)	6.78 (1.19)	< 0.001
Hospital LOS	18 (10–30)	19 (10–33)	0.43
ICU LOS	10 (5–17)	11 (6–18)	0.04
Ventilator days	7 (3–14)	9 (5–15)	0.01
Mortality	224 (27.3)	120 (38.7)	< 0.001

*Abbreviations*: *ARDS* acute respiratory distress syndrome, *APACHE* Acute Physiologic and Chronic Health Evaluation, *SOFA* Sequential Organ Failure Assessment, *ICU* intensive care unit, *PEEP* positive end-expiratory pressure, *PBW* predicted body weight, *LOS* length of stay

Data presented as mean (SD), median (IQR), or *n* (%)

^a^May have more than one admitting diagnosis. Does not sum to 100%

**Fig. 2:**
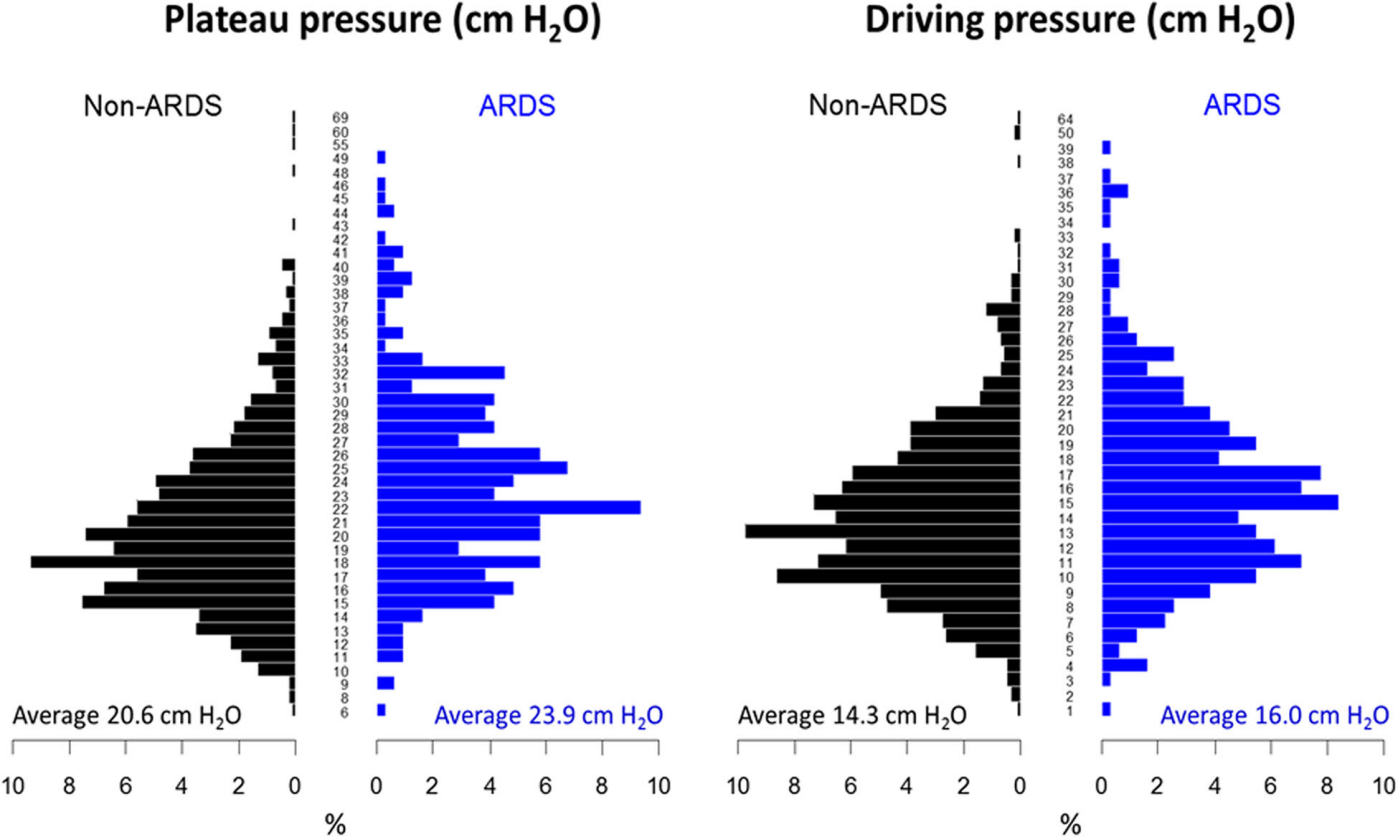
Distribution and average Pplat and ΔP by ARDS status. *p value for the difference in plateau pressure and ARDS status is < 0.001. **p value for the difference in driving pressure and ARDS status is < 0.001

**Fig. 3:**
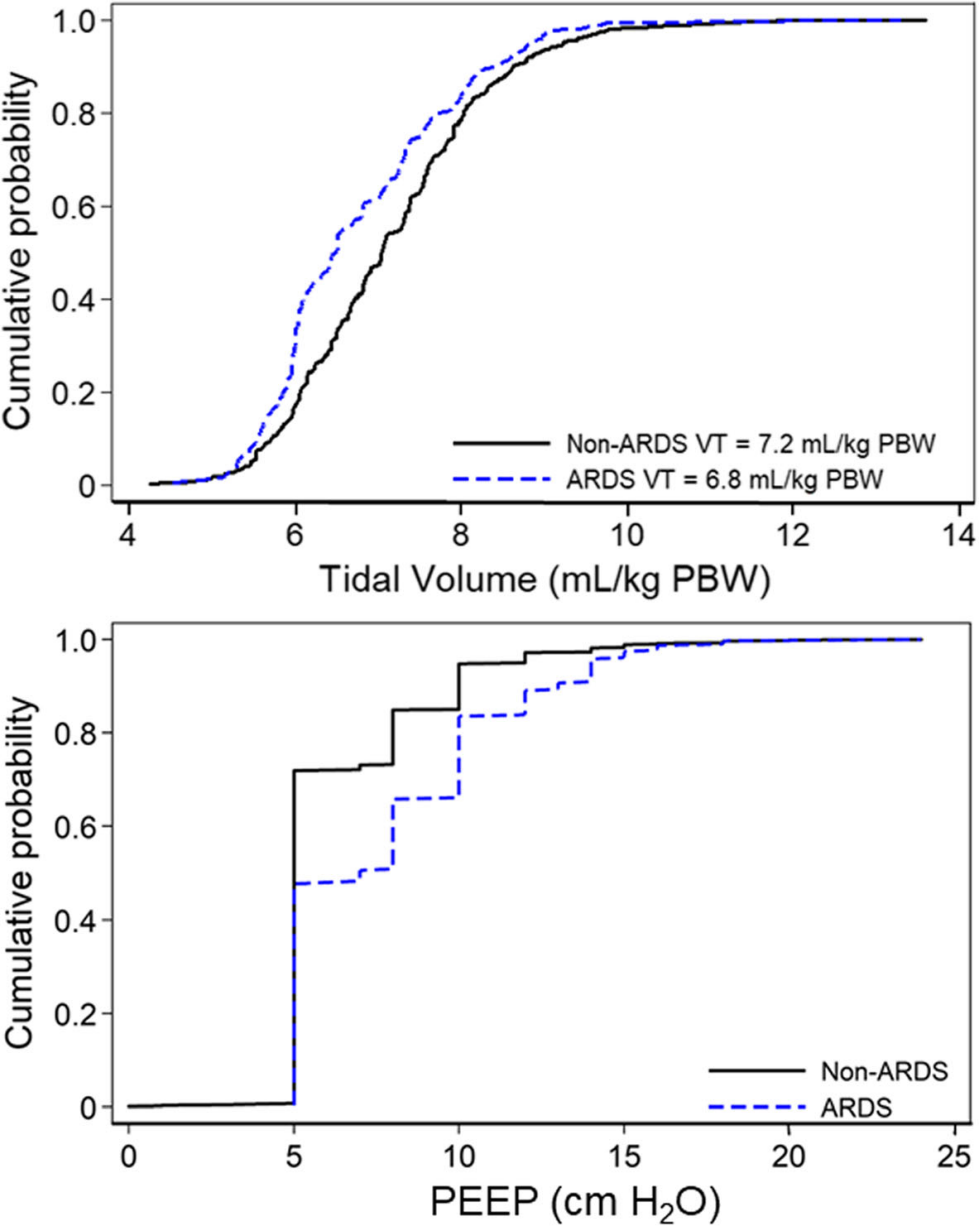
Cumulative distribution of tidal volume and PEEP by ARDS status. *p value for a difference in means of tidal volume for ARDS vs. non-ARDS is < 0.001. **p value for a difference in means of PEEP for ARDS vs. non-ARDS is < 0.001

Average Pplat and ΔP were lower in participants without ARDS (Pplat 20.6 vs. 23.9 cm H_2_O, *p* <  0.001; and ΔP 14.3 vs. 16.0 cm H_2_O, *p* <  0.001) as compared to those with ARDS (Fig. 2). Non-ARDS participants received significantly higher tidal volumes (7.2 vs. 6.8 mL/kg predicted body weight (PBW); *p* <  0.001) and significantly lower PEEP (5 vs. 7 cm H_2_O; *p* <  0.001) than ARDS participants (Fig. 3).

There were more participants enrolled from medical ICUs (*n* = 539) compared to the surgical (*n* = 298) or mixed ICUs (*n* = 295). Participants in the medical ICUs received significantly lower tidal volumes per kilogram PBW compared to the surgical and mixed ICUs (see Additional file 1: Table S1). However, there were no differences in ΔP or Pplat between types of ICUs. Participants in surgical ICUs had higher days on mechanical ventilation and length of stay; however, they had lower mortality rates.

### Ventilator pressures and mortality

**Fig. 4:**
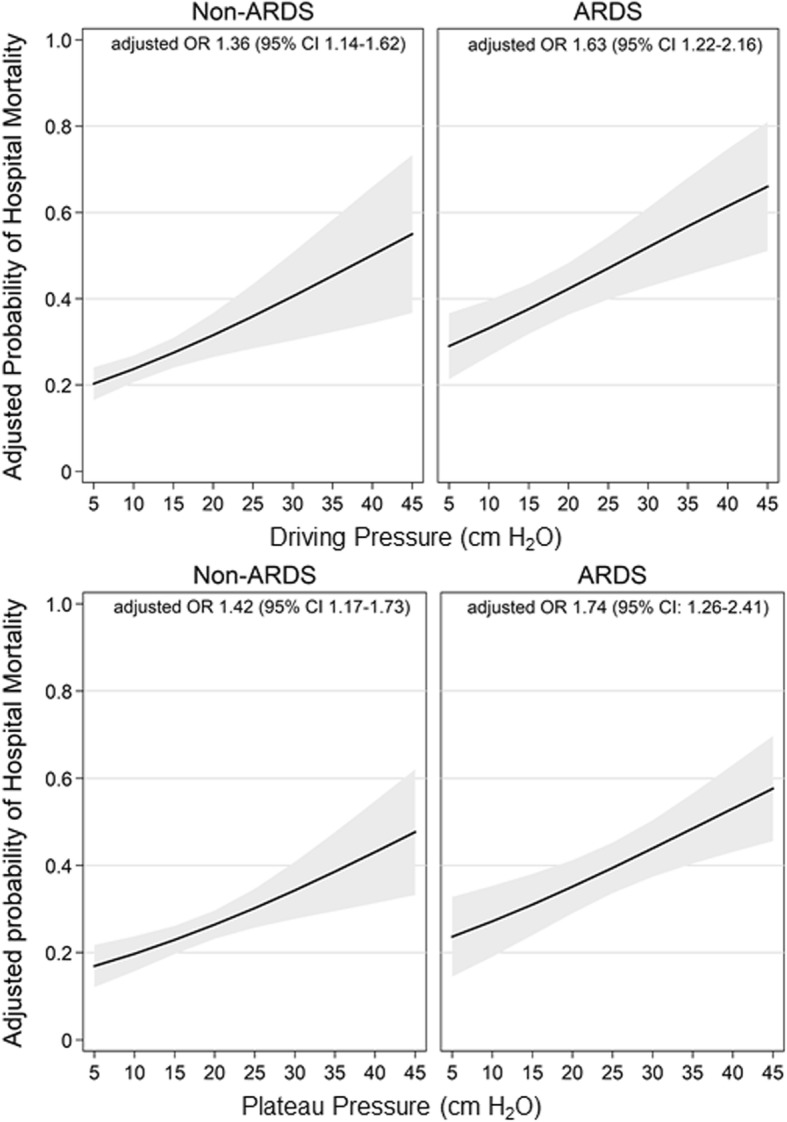
Predicted probability of hospital mortality by increases in driving pressure and plateau pressure by ARDS status. Adjusted OR for mortality is scaled per IQR increment of ΔP (7 cm H_2_O) and Pplat (8 cm H_2_O), and adjusted for age, sex, APACHE II, vasopressor use, sepsis, hospital volume, and ICU category

In unadjusted and adjusted analyses, ΔP was independently associated with hospital mortality in both non-ARDS and ARDS participants. After adjusting for covariates, non-ARDS participants demonstrated an increased odds of hospital mortality per IQR increment of ΔP (adjusted OR = 1.36 per 7 cm H_2_O, 95% CI 1.14–1.62) and per IQR increment of Pplat (adjusted OR = 1.42 per 8 cm H_2_O, 95% CI 1.17–1.73) (Table 2). Similarly, ARDS participants demonstrated an increased odds of mortality per IQR increment of ΔP (adjusted OR = 1.63 per 7 cm H_2_O, 95% CI 1.22–2.16) and Pplat (adjusted OR = 1.74 per 8 cm H_2_O, 95% CI 1.26–2.41) (Fig. 4). Higher APACHE II scores were also associated with mortality in both populations, while use of vasopressors was associated with mortality only in participants without ARDS. The presence of sepsis, hospital volume, and type of ICU were not associated with survival in either group.

**Table 2:** Odds of hospital mortality from multivariable logistic regression

	Non-ARDS			ARDS		
	OR^a^	95% CI	*p* value	OR^a^	95% CI	*p* value
Driving pressure (per 7 cm H_2_O)^b^	1.36	1.14–1.62	< 0.001	1.63	1.22–2.16	< 0.001
Plateau pressure (per 8 cm H_2_O)^b^	1.42	1.17–1.73	< 0.001	1.74	1.26–2.41	< 0.001
Age (per 5 years)	1.05	0.98–1.11	0.125	1.09	0.99–1.20	0.07
PEEP (per 1 cm H_2_O)	1.05	0.98–1.11	0.16	1.12	1.07–1.17	< 0.001
APACHE II (per 1 point)	1.08	1.04–1.11	< 0.001	1.08	1.04–1.12	< 0.001
Vasopressor use	1.52	1.06–2.16	0.02	1.02	0.56–1.85	0.94
Sepsis	1.12	0.77–1.62	0.56	1.03	0.62–1.69	0.90

*Abbreviations*: *ARDS* acute respiratory distress syndrome, *APACHE* Acute Physiologic and Chronic Health Evaluation, *PEEP* positive end-expiratory pressure, *OR* odds ratio

Estimates for covariates are derived from the driving pressure model. The plateau pressure model included the same covariates as the driving pressure model

^a^Odds ratio adjusted for age, sex, PEEP, APACHE II, vasopressor use, sepsis, hospital volume, and ICU category

^b^Odds ratios for driving pressure and plateau pressure are scaled to IQRs

### Sensitivity analyses

There were no significant interaction effects between ΔP and ARDS status (*p* = 0.32 from likelihood ratio test) or between Pplat and ARDS status (*p* = 0.13 from likelihood ratio test) on hospital mortality. Our results were robust to restricting the range of Pplat to less than 35 cm H_2_O, and ΔP remained independently associated with hospital mortality in non-ARDS participants in this population. Similarly, ΔP remained independently associated with hospital mortality in non-ARDS participants when we excluded participants with PEEP < 5 cm H_2_O (*n* = 4). Our results were also robust to restricting the participant population to those on controlled modes of ventilation (see Additional file 1: Tables S2, S3, and S4). We were unable to fully assess for interactions between airway pressures and the presence of spontaneous breathing as we did not have information on the actual total respiratory rate compared to the set respiratory rate of patients on controlled modes of ventilation. Finally, ΔP remained independently associated with hospital mortality in non-ARDS participants in both hypoxemic respiratory failure (PF < 300 mmHg, adjusted OR = 1.41, 95% CI 1.08–1.84 per 7 cmH_2_O) and non-hypoxemic respiratory failure (PF ≥ 300 mmHg, adjusted OR = 1.38 per 7 cmH_2_O, 95% CI 1.08–1.75).

## Discussion

The results of this study suggest that monitoring and potentially limiting inspiratory airway pressures is important for all mechanically ventilated patients, not just patients with ARDS. In our multicenter prospective observational cohort, we demonstrate that inspiratory airway pressures were independently associated with hospital mortality in a cohort of non-ARDS participants requiring mechanical ventilation. We also confirm the previously reported association between inspiratory airway pressures and mortality in ARDS participants [5–8, 16, 17]. Our results are consistent with prior meta-analyses and epidemiologic studies demonstrating improved outcomes from low versus higher tidal volume ventilation and lower ΔP and Pplat in patients without ARDS [9, 18–20]. In our study, tidal volumes on average at time of study enrollment were within the target range of 6–8 mL/kg PBW [4]. Even within this range of tidal volumes, however, lower ΔP was associated with increased survival.

Our results suggest that higher ΔP and Pplat reflect potentially injurious stresses on the lungs of non-ARDS patients that are associated with higher odds of mortality. ΔP is determined by the distribution of a tidal volume across the available aerated lung (represented by respiratory system compliance). Patients without ARDS may benefit from reductions in ΔP to prevent or mitigate ventilator-induced lung injury. For example, patients with unilateral pneumonia will have a heterogeneous distribution of tidal volume based on the reduced volume of aerated lung. Increased ΔP in this type of patient may indicate the need for further reductions in tidal volume to prevent overdistention of the unilaterally aerated lung. Similarly, patients with obstructive airways disease may have a heterogeneous distribution of tidal volume and differences in regional transpulmonary pressures due to variations in emptying time of different lung regions (i.e., time constant). In these patients, changes in ΔP could provide information on the influence of different PEEP levels on airflow limitations. Thus, even without the diagnosis of ARDS, ΔP may convey important clinical and prognostic information for the patient with acute respiratory failure. Additionally, even patients without a primary lung disease may benefit from limiting ΔP. Neto and colleagues demonstrated an increased risk of postoperative complications with higher ΔP in patients with normal lungs undergoing surgery [9]. We suggest future clinical trials evaluate whether implementing lung-protective ventilation strategies to limit ΔP increases survival for all mechanically ventilated patients, with or without ARDS.

In contrast, a recent retrospective study by Schmidt and colleagues suggested that ΔP was not associated with mortality in non-ARDS patients [10]. Although their study was similarly powered to ours, it was a single-center cohort and ARDS status was ascertained retrospectively. Furthermore, they had with a limited range of Pplat and PEEP, which may have attenuated the association between ΔP and mortality in their study. In our study, the non-ARDS participants had a wider range of ΔP (interquartile range 10–17 cmH_2_O) and lower respiratory system compliance (39.6 L/cmH_2_O) compared to the participants in the Schmidt study [10]. Additionally, the Schmidt study included a non-ARDS study population with a low incidence of a pulmonary indication for ICU admission. Their sample had a 4% incidence of pneumonia. In our study, 50.6% of participants had an initial ICU admitting diagnosis related to the respiratory system, with a 30.4% incidence pneumonia. It is possible that the higher incidence of primary lung pathology and subsequent increased risk for ventilator-induced lung injury in our study, as well as the broader range of ΔP and Pplat, improved the power of our study to identify a significant relationship between ΔP and mortality.

Recently, the PReVENT trial investigated a low tidal volume (goal 6 mL/kg PBW) versus an intermediate tidal volume (goal 10 cc/kg PBW) in patients without ARDS. The investigators failed to find a difference in patient outcomes between a low and intermediate tidal volume strategy in non-ARDS patients [18]. However, in this study, baseline ΔP in both groups was relatively low and changed minimally with the tidal volume intervention (low tidal volume group baseline to day 1 ΔP, 11 to 10 cmH_2_O; intermediate tidal volume group baseline to day 1 ΔP, 13 to 13 cmH_2_O). Consistent with the mediation analysis performed by Amato and colleagues [5], the change in driving pressure may be more important than the change in tidal volume in terms of reducing mortality in mechanically ventilated patients. Additionally, by day 1 following randomization, a majority of participants in the low tidal volume group received pressure support ventilation, which permitted large spontaneous tidal volumes outside of the target 6 mL/kg PBW. The results of the PReVENT trial highlight the importance of prospectively evaluating alternate lung-protective mechanical ventilation strategies in the non-ARDS population. We suggest future trials should focus on limiting ΔP rather than on targeting an absolute tidal volume goal.

Our study has several strengths. First, it is a large prospective cohort study with data from 59 different ICUs, both medical and surgical, geographically dispersed across the USA, which increases the generalizability of our findings. Data were collected prospectively by experienced and trained research coordinators. ARDS status was ascertained prospectively and similarly to how it is determined in a clinical setting. Second, our participants demonstrated wide ranges of ΔP, Pplat, and PEEP, which allow for evaluation across the spectrum of severity of respiratory failure. Third, we internally validated our dataset by evaluating the association between ΔP and ARDS in addition to analyzing the relationship in non-ARDS participants. Finally, our findings are in concordance with other epidemiologic studies suggesting that ΔP is associated with clinical outcomes in non-ARDS participants [9, 19].

Although our results are significant, our study has some potential shortcomings. One major limitation is that our values for Pplat and ΔP were not always derived from the same day of the ICU course across all of the participants. Some participants were enrolled into the study on ICU day 1, others on day 5 and between, and we do not know this distribution of time to intubation to enrollment, which precludes further sensitivity analysis. Needham and colleagues described the importance of timing of lung-protective ventilation and showed that there was a large mortality benefit in starting this earlier rather than later [21]. Misclassification bias could potentially limit our findings as well. Based on enrollment timing, some of the non-ARDS participants may have already starting to develop ARDS contributing to misclassification bias. Alternatively, some of the non-ARDS participants may have developed ARDS after study enrollment, which could have overestimated our non-ARDS mortality and underestimated ARDS mortality. Finally, although ARDS status was prospectively ascertained by trained research coordinators, the diagnosis of ARDS is routinely missed by seasoned clinicians [16, 22]. A sensitivity analysis, however, confirmed that our findings were robust even when classifying participants by the presence or absence of hypoxemic respiratory failure (PaO_2_/FiO_2_ < 300) rather than diagnosis of ARDS. Nevertheless, many of our non-ARDS participants were relatively hypoxemic with reduced compliance suggesting their lung disease was severe despite the lack of ARDS. Future studies confirming our results will need to standardize the time of airway pressure measurement and consider the longitudinal effect of airway pressures on the risk for mortality and ARDS development.

Another limitation is that the majority of these ICUs were academic which can limit the generalizability of our findings. Furthermore, there were a large number of mechanically ventilated patients who did not have Pplat measured and were excluded from our study. Because our protocol specified obtaining Pplat on all patients if possible, the included patients are presumably different than those who had Pplat measured. Additionally, Pplat and ΔP reflect both lung and chest wall compliance and are potentially affected by spontaneous respiratory efforts. In patients who are spontaneously breathing or have altered chest wall compliance, airway pressures are imperfect measures of lung stress, although may still be adequate surrogates [7]. Finally, our study does not prove a causal effect between lower ΔP and improved survival in non-ARDS participants. Respiratory system compliance, a major component of ΔP, is a strong independent predictor of mortality. The complex mathematical and physiologic coupling between ΔP, compliance, tidal volume, and PEEP further complicates efforts to confirm a causal relationship between ΔP and mortality. However, our results add significantly to the body of evidence supporting the need for randomized controlled trials comparing ΔP-targeted ventilation strategies compared to our current practice of limiting tidal volumes only.

## Conclusions

Our findings demonstrate that greater ΔP and Pplat are associated with increased mortality in patients without ARDS, similar to the findings in patients with ARDS. In mechanically ventilated patients, ΔP and Pplat may be useful markers of lung stress. ΔP and Pplat demonstrated a linear relationship with odds of mortality, suggesting that lower ΔP and Pplat are better. Recent evidence suggests ΔP may be a critical target for mechanical ventilation in ARDS patients. ΔP can be lowered by reducing tidal volume or by optimizing respiratory system compliance through PEEP titration or proning [23–25]. Future research should prospectively evaluate the feasibility and efficacy of ventilation strategies that limit ΔP. Based on our results, these ventilation strategies should be evaluated in both ARDS and non-ARDS patient populations.

## Supplementary information

**Additional file 1 Table S1.** Shows baseline characteristics of participants by type of ICU. **Table S2.** Shows baseline characteristics of participants by controlled versus spontaneous mode of ventilation. **Table S3.** Shows the proportion of participants receiving each ventilator mode. **Table S4.** Shows the multivariable logistic regression results from the sensitivity analysis of participants receiving controlled ventilator modes.

## Data Availability

The datasets used and/or analyzed during the current study are available from the corresponding author on reasonable request.

## Abbreviations

ARDS: Acute respiratory distress syndrome
Pplat: Plateau pressure
ΔP: Driving pressure
PEEP: Positive end-expiratory pressure
ICU: Intensive care unit
LOS: Length of stay
PBW: Predicted body weight
SCCM: Society of Critical Care Medicine
CIOS: Critical Illness Outcomes Study
APACHE: Acute Physiologic and Chronic Health Evaluation
SOFA: Sequential Organ Failure Assessment
