# Supplementary material for: Association between hospital mortality and inspiratory airway pressures in mechanically ventilated patients without acute respiratory distress syndrome: a prospective cohort study
Source: Crit Care. 2019 Nov 21;23:367. doi: 10.1186/s13054-019-2635-y (PMC6868689; doi:10.1186/s13054-019-2635-y)
Supplement: Supplementary file 1 — Additional file 1 Table S1. Shows baseline characteristics of participants by type of ICU. Table S2. Shows baseline characteristics of participants by controlled versus spontaneous mode of ventilation. Table S3. Shows the proportion of participants receiving each ventilator mode. Table S4. Shows the multivariable logistic regression results from the sensitivity analysis of participants receiving controlled ventilator modes. [file 13054_2019_2635_MOESM1_ESM.docx]

**Table S1. Baseline Characteristics by ICU Type**

|  | **Medical ICU**  **(n=539)** | **Surgical ICU**  **(n=298)** | **Mixed ICU**  **(n=295)** |
| --- | --- | --- | --- |
| Age | 60.4 (16.0) | 58.2 (17.7) | 61.1 (16.1) |
| Female | 256 (47.5) | 126 (42.3) | 140 (47.5) |
| Presence of ARDS | 164 (30.4) | 48 (16.1) | 98 (33.2) |
|  |  |  |  |
| Plateau Pressure (Non-ARDS) | 21.2 (6.9) | 20.3 (6.2) | 20.0 (6.1) |
| Plateau Pressure (ARDS) | 23.8 (7.2) | 24.8 (8.0) | 23.5 (6.4) |
|  |  |  |  |
| Driving Pressure (Non-ARDS) | 14.8 (6.4) | 13.7 (5.6) | 14.1 (5.5) |
| Driving Pressure (ARDS) | 15.7 (6.6) | 16.5 (6.8) | 16.5 (6.8) |
|  |  |  |  |
| Tidal Volume (mL/kg PBW) | 6.6 (1.07) | 7.52 (1.21) | 7.38 (1.26) |
|  |  |  |  |
| Mortality | 172 (31.9) | 73 (24.3) | 99 (33.6) |
| Days on Ventilator | 8 (4-13) | 9 (3-19) | 7 (3-15) |
| ICU LOS | 10 (5-16) | 13 (6-23) | 10 (5-16) |
| Hospital LOS | 16 (9-28) | 21.5 (12-34) | 18 (10-31) |

Abbreviations: ARDS, Acute Respiratory Distress Syndrome; ICU, Intensive Care Unit; PBW, Predicted Body Weight; LOS, Length of Stay

^a^Data presented as mean (SD), median (IQR), or n (%)

**Table S2: Baseline Characteristics by Controlled versus Spontaneous Mode of Ventilation of Non-ARDS Participants**

|  | **Controlled MV**^b^ **(n=605)** | **Spontaneous MV**^b^ **(n=217)** | **p-value** |
| --- | --- | --- | --- |
| Age (years) | 59.3 (16.9) | 63.2 (15.7) | 0.003 |
| Male | 288 (47.6) | 94 (43.3) | 0.28 |
| Admitting Diagnosis^c^ |  |  |  |
| Respiratory | 344 (56.9) | 72 (33.2) | <0.001 |
| Neurological | 162 (26.8) | 89 (41.0) | < 0.001 |
| Cardiovascular | 177 (29.3) | 49 (22.6) | 0.06 |
| Infectious | 169 (27.9) | 52 (24.0) | 0.26 |
| Gastrointestinal | 89 (14.7) | 33 (15.2) | 0.86 |
| Trauma | 43 (7.1) | 18 (8.3) | 0.57 |
| Endocrine | 25 (4.1) | 13 (6.0) | 0.26 |
| Other | 106 (17.5) | 28 (12.9) | 0.11 |
| APACHE II | 20.1 (7.3) | 20.2 (7.4) | 0.87 |
| SOFA | 6 (4 - 9) | 6 (4 - 9) | 0.20 |
| Plateau Pressure | 20.8 (6.5) | 20.1 (6.5) | 0.16 |
| Driving Pressure | 14.4 (5.9) | 14.1 (6.1) | 0.52 |
| PEEP | 5 (5 - 8) | 5 (5 - 5) | 0.012 |
| Tidal Volume (mL/kg PBW) | 6.8 (6.1 – 7.7) | 7.6 (6.8 – 8.0) | < 0.001 |
| Hospital LOS | 18 (10 – 30) | 18 (9 – 31) | 0.87 |
| ICU LOS | 10 (5 – 16) | 10 (5 – 18) | 0.61 |
| Ventilator Days | 8 (3 - 14) | 7 (3 – 13) | 0.46 |
| Mortality | 156 (25.8) | 68 (31.3) | 0.12 |

^a^Data presented as mean (SD), median (IQR), or n (%)

^b^Controlled Mechanical Ventilation Mode includes: Volume Control, Synchronized Intermittent Mandatory Ventilation (SIMV), Pressure Control, and Pressure-Regulated Volume Control. Spontaneous Modes of Ventilation include: Pressure Support, Airway Pressure Release Ventilation (APRV), or Other.

^c^May have more than one admitting diagnosis. Does not sum to 100%

**Table S3: Mode of Ventilation for Non-ARDS Participants:**

| **Mode (n=822)** | **N (Percent)** |
| --- | --- |
| Volume – Control | 450 (54.7) |
| Synchronized Intermittent Mandatory Ventilation (SIMV) | 85 (10.3) |
| Pressure – Control | 8 (1.0) |
| Pressure Regulated Volume Control (PRVC) | 62 (7.5) |
| Pressure Support | 44 (5.4) |
| Airway Pressure Release Ventilation (APRV) | 2 (0.2) |
| Other | 171 (20.8) |

**Table S4:** **Odds of hospital mortality for non-ARDS participants from multivariable logistic regression by controlled or spontaneous ventilator mode**

|  |  | **Driving Pressure**  **(per 7 cm H2O)^b^** | | |
| --- | --- | --- | --- | --- |
|  |  | **OR^a^** | **95% CI** | **p-value** |
| Total Non-ARDS population |  | 1.36 | 1.14-1.62 | <0.001 |
| ^c^Controlled Modes |  | 1.39 | 1.14-1.72 | <0.001 |
| ^d^Spontaneous modes |  | 1.36 | 1.00-1.86 | <0.05 |

^a^Odds ratio adjusted for age, sex, PEEP, APACHE II, vasopressor use, sepsis, hospital volume, ICU category

^b^Odds ratio for Driving Pressure are scaled to IQR

^c^Controlled Mechanical Ventilation Mode includes: Volume Control, Synchronized Intermittent Mandatory Ventilation (SIMV), Pressure Control, and Pressure-Regulated Volume Control.

^d^Spontaneous Modes of Ventilation include: Pressure Support, Airway Pressure Release Ventilation (APRV), or Other.

**Table S5: Association between Respiratory System Compliance and Mortality in Non-ARDS Participants**

|  |  | **Compliance Respiratory System** | | |
| --- | --- | --- | --- | --- |
|  |  | **OR** | **95% CI** | **p-value** |
| Univariate |  | 0.98 | 0.98 – 0.99 | 0.011 |
| ^a^Multivariate |  | 0.99 | 0.98 – 1.0 | 0.039 |

^a^Odds ratio adjusted for age, sex, PEEP, APACHE II, vasopressor use, sepsis, hospital volume, ICU category
